# Reply: Let's talk about our grade

**DOI:** 10.1016/j.xjon.2025.08.004

**Published:** 2025-08-22

**Authors:** Toyokazu Endo, Victor van Berkel

**Affiliations:** Department of Cardiovascular and Thoracic Surgery, University of Louisville School of Medicine, Louisville, Ky

To the Editor:

**Figure:**
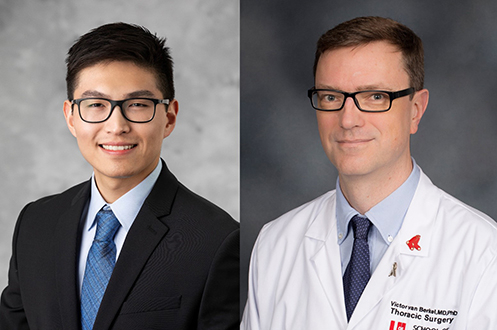

It is a pleasure that our original manuscript has invited a scholarly discussion regarding the histologic grades for non–small cell lung cancer, specifically that of adenocarcinoma.^1^ In our original manuscript, we used the National Cancer Database (NCDB) to identify all patients who had undergone a primary surgical resection of lung adenocarcinoma.^2^ As mentioned in our limitations section, it is unfortunate that there were not enough descriptions regarding the histologic features beyond simply denoting them as “well-differentiated,” “moderately differentiated,” and “poorly differentiated/undifferentiated.”

As Pohlman and McMullen have pointed out in their letter, the International Association for the Study of Lung Cancer has proposed a new classification for nonmucinous adenocarcinoma.^1^^,^^3^ The proposal's goal was to unify the current grading system, provide prognostic data, and potentially aid in emerging management and treatment. One key limitation to the proposal set out by the committee is that the dataset used to construct the grading system, although multi-institutional, requires further validation. Although the NCDB is limited in its histologic data, it does include all patients across the United States who have been treated at a cancer center accredited by the American College of Surgeons, thus furthering the argument that regardless of how the adenocarcinoma was graded, it provides prognostic value that cannot be ignored. Despite this, the ninth edition of the TNM classification, published this year, does not include grade as part of the staging workup.^4^

Pohlman and McMullen also emphasize that the future of precision medicine and genomic testing to assist in treatment is approaching. However, until this becomes the standard of care for patients with low-stage adenocarcinoma, other methods will still be necessary to improve patient care. In our study, we included patients from 2013 to 2020. The proposed classification by the International Association for the Study of Lung Cancer was introduced in 2020, and various methods were likely used to grade the cancer. The 2015 World Health Organization classification also notes that multiple grading methods exist, ranging from architectural to nuclear approaches, and it is unclear which grading system was used in the NCDB.^5^ Regardless of the grading method used, knowing the histology grade for stage 1 lung adenocarcinoma can help inform future treatments. With the new classification system, we hope to achieve a more consistent approach to grading lung cancer. We thank the authors for providing us with another avenue to further our discussion on how we can further improve survival in these patients using data that may be overlooked.

## Conflict of Interest Statement

Dr van Berkel is the CMO and Co-Founder of Breath Diagnostics Inc. Dr Endo reported no conflicts of interest.

The *Journal* policy requires editors and reviewers to disclose conflicts of interest and to decline handling or reviewing manuscripts for which they may have a conflict of interest. The editors and reviewers of this article have no conflicts of interest.
